# Similarly to *BmToll9-1*, *BmToll9-2* Is a Positive Regulator of the Humoral Immune Response in the Silkworm, *Bombyx mori*

**DOI:** 10.3390/insects15121005

**Published:** 2024-12-19

**Authors:** Jisheng Liu, Weijian Chen, Sihua Chen, Shuqiang Li, Luc Swevers

**Affiliations:** 1 School of Life Sciences, Guangzhou University, Guangzhou 510006, China; 2 Institute of Biosciences and Applications, National Centre for Scientific Research Demokritos, 15431 Athens, Greece; swevers@bio.demokritos.gr

**Keywords:** *Bombyx mori*, *BmToll9-2*, Toll receptor, antimicrobial peptides, immune response, *Escherichia coli*

## Abstract

Toll receptors play important roles in the development and innate immunity of insects. Previously, we reported the immunological function of *BmToll9-2* in silkworm, *Bombyx mori*, larvae. In this study, we focused on the role of *BmToll9-2* as a regulator in the Toll signaling pathway. The expressions of most signaling genes in the Toll pathway, as well as immune effectors, were reduced after the RNAi of *BmToll9-2*. Coincidentally, hemolymph from *BmToll9-2*-silenced larvae exhibited decreased antibacterial activity in the growth of *Escherichia coli*, demonstrated either by growth curve or inhibitory zone experiments. The oral administration of heat-inactivated *E. coli* and *Staphylococcus aureus* following the RNAi of *BmToll9-2* up-regulated the expression of most signaling genes in the Toll pathway and downstream immune effectors. The above results indicate that *BmToll9-2* is positively involved in the Toll signaling pathway. As a positive regulator, *BmToll9-2* is shown to be activated preferentially against *E. coli* and, in turn, positively modulates the humoral immune response in antibacterial activity.

## Simple Summary

A deep understanding of the role of Toll receptors in the innate immunity of insects is mostly restricted to the model insect *Drosophila melanogaster*, and much more research is needed to reveal their role in other insects. This article focuses on the *BmToll9-2* receptor, one of 14 Toll-related receptors in the silkworm, *Bombyx mori*. Similarly to its closely related paralog, *BmToll9-1*, *BmToll9-2* acts as a positive regulator of the innate humoral response in the midgut of silkworm larvae, as evidenced by RNAi and bacterial challenge experiments. More specifically, the expression level of *BmToll9-2* is positively correlated with those of intermediary genes in the Toll signaling pathway as well as with immune effector genes, such as antimicrobial peptides. Consequently, both Toll9 receptors in the silkworm, *BmToll9-1* and *BmToll9-2*, act redundantly to stimulate antibacterial activity and immune-related gene expression in the Toll pathway.

## 1. Introduction

Insects have existed on Earth for more than 350 million years, preceding the age of dinosaurs and the emergence of flowering plants. Therefore, they have evolved powerful immunity. Unlike mammals that mainly depend on adaptive immunity, insects only have innate immunity which provides the first line of defense against invading pathogens such as viruses, bacteria, and fungi [1]. In general, innate immunity in insects comprises cellular and humoral immunity. Cellular immunity is directly mediated by hemocytes (e.g., phagocytosis, nodulation, and encapsulation). Humoral immunity consists of the synthesis of antimicrobial peptides (AMPs) to defend against pathogens, which is regulated by several immune signaling pathways such as Toll, immune deficiency (IMD), Janus kinase/signal transducers and activators of transcription (Jak/Stat), and other pathways [2]. Among these immune signaling pathways, the Toll pathway is triggered after the ligand Spätzle binds to Toll [3]. Therefore, Toll receptors function as bridges to connect signaling transduction from the outside to the inside of cells.

Toll was originally identified in *Drosophila melanogaster* as a gene controlling dorsal–ventral polarity during embryo development [4]. It was later found to be a transmembrane receptor [5] and involved in anti-fungal immunity [2]. Another eight Toll genes were identified in subsequent studies and proved to function in the innate immune response in *Drosophila* [6]. *Drosophila* Toll receptors are not typical pattern-recognition receptors (PRRs) that directly recognize pathogen-associated molecular patterns (PAMPs) to regulate the innate immune defense [7]. Several downstream factors are involved in the Toll signaling pathway, including MyD88, Tube, Pelle, Cactus, and Rel. Intercellular components such as Tollip, Pellino, TNF receptor-associated factor-2 (TRAF2), and ECSIT are also involved in the Toll signaling pathway [8]. Among them, Tollip is a Toll-interacting protein that is a critical regulator of Toll-like receptor (TLR)-mediated innate immune responses.

The silkworm, *Bombyx mori*, is a domesticated insect in China with economic importance in agriculture. It is the first lepidopteran insect to have its genome sequenced and published in a public database [9], which confirmed the silkworm as a model insect in studies for gene functions and innate immune responses in Lepidoptera. Regarding Toll signaling pathways, 14 Toll-related genes have been identified in the *B. mori* genome, which are phylogenetically divided according to functions related to immunity and various other processes [8,10]. Although there are 14 Toll-related genes present in silkworms, current publications are focused on the Toll9 gene, despite the fact that Toll7 was the first Toll gene that was cloned [11] (Table 1).

In immunity-related Toll receptors, Toll9 is a conserved receptor which is well studied for its role in immunity. Two Toll9 receptors, *BmToll9-1* and *BmToll9-2*, are present in the silkworm. *BmToll9-1* was the first reported Toll9 receptor, which is abundant in the gut to respond to the invasion of different microbial pathogens [12]. We previously reported that the transcription of *BmToll9-1* was reduced after lipopolysaccharide (LPS) or double-stranded RNA (dsRNA) was injected into silkworm larvae [13]. We also found that LPS inhibited AMP genes and genes in the IMD and Jak/Stat pathways in silkworm-derived Bm5 cells overexpressing the *BmToll9-1* receptor [14]. *BmToll9-1* was induced by *Beauveria bassiana* challenge [15]. The knockdown of *BmToll9-1* led to a higher accumulation of nuclear polyhedrosis virus (BmNPV) [16]. A two-hybrid experiment on yeast showed that *BmToll9-1* could interact with BmSpz2, which in turn induced the expression of AMPs [17]. *BmToll9-1* was later proved to be a PRR for the LPS signaling pathway [18]. RNA interference (RNAi) has evolved into a potent tool for gene functional studies in fundamental research, even though RNAi in silkworms is weak compared to that in other orders of insects. However, the first successful lepidopteran RNAi report was the knockdown of a pigment gene in *Bombyx* [21], which suggests that RNAi is feasible in silkworms. A comprehensive analysis in Lepidoptera showed that immunity-related genes had a higher RNAi efficiency [22]. In our recent report, the successful knockdown of *BmToll9-1* resulted in smaller and lighter larvae and cocoons. Meanwhile, *BmToll9-1* positively regulated the downstream signaling and AMP genes, which caused increased antibacterial activity against *Escherichia coli* [19]. Although *BmToll9-1* and *BmToll9-2* are phylogenetically close to each other, studies on *BmToll9-2* are far fewer in comparison [20]. Meanwhile, both *BmToll9-1* and *BmToll9-2* are two distinct genes. The role of *BmToll9-2* cannot be presumed based on the work on *BmToll9-1*. It can only be confirmed by experimental data.

Previously, we characterized the immunological function of *BmToll9-2* in the silkworm larval midgut. *BmToll9-2* was activated by *E. coli* and LPS. The RNAi-mediated knockdown of *BmToll9-2* reduced the weight and growth of the silkworm. Bacterial challenge following RNAi up-regulated the expression of *BmToll9-2* and rescued the weight differences in the silkworm [20]. In this follow-up study, we focus on the role of *BmToll9-2* in regulating the downstream genes in the Toll pathway, which positively modulates the humoral immune response and antibacterial activity in hemolymph.

## 2. Materials and Methods

### 2.1. Insect Rearing and Bacterial Culture

*B. mori* silkworms were provided by Guangdong Academy of Agricultural Sciences. The larvae were reared daily on fresh mulberry leaves and cultured at 25 ± 1 °C, with a photoperiod of 12 L:12 D, with a relative humidity of 75 ± 5%. The bacteria *E. coli* (Gram-negative) and *Staphylococcus aureus* (Gram-positive) were maintained in our laboratory, which were inoculated at 37 °C for 2–3 h in Luria–Bertani (LB) liquid medium until the OD_600_ = 0.5 was achieved prior to use.

### 2.2. RNAi Protocol and Bacterial Challenge to Larvae

A detailed protocol to silence the target gene in the silkworm larvae was described in our recent publication [20]. More studies on RNAi in silkworms can be found in recent publications [23,24]. Briefly, dsRNA corresponding to *BmToll9-2* (dsBmToll9-2) was generated with the T7 RiboMAX Express RNAi System (Promega). RNAi was performed by injecting dsBmToll9-2 into 5th-instar larvae between the 2nd and 3rd abdominal segment on day 1 after molting (N = 50 per treatment for each repeat). The negative control consisted of injecting dsRNA corresponding to green fluorescent protein (GFP). The midgut samples were collected 24 h after the RNAi treatment for subsequent analysis.

The larvae, after the RNAi above, were subjected further to bacterial challenge. The bacteria were centrifuged at 8000× *g* for 5 min. The cell pellets were resuspended with sterile water at roughly 5 × 10^8^ cells/mL and then heated at 80 °C for 5 min. Each larva was provided with the same size of a mulberry leaf disk coated with 10 μL of bacterial solution. The midgut samples were collected 24 h after the feeding of bacteria for subsequent analysis.

### 2.3. RNA Extraction and cDNA Synthesis

Total RNA was extracted from midgut tissue using RNAiso Plus (TaKaRa, Kusatsu, Japan). The RNA was then digested with RNase-free DNase I (TaKaRa) to remove the genomic DNA. Complementary DNA (cDNA) was synthesized with the PrimeScript RT Reagent Kit (Perfect Real Time) (TaKaRa) using Oligo dT and Random 6-mers as primers. The cDNA was used in subsequent qPCR reactions.

### 2.4. Quantitative Real-Time PCR (qPCR)

The qPCR reaction was performed using the GoTaq qPCR Master Mix (Promega, Madison, WI, USA). Thermal amplification was performed with the CFX Connect Real-Time System (Bio-Rad, Hercules, CA, USA) as follows: 95 °C for 2 min, 40 cycles of 95 °C for 15 s, and 57 °C for 30 s. Each signaling gene in the signaling pathway and each representative effector gene were selected [8]. Primers were designed to detect the signaling genes and effector genes. Translation initiation factor 4A (BmTIF4A) and translation initiation factor 3 subunit 4 (BmTIF3s4) were used as reference genes (Table 2). The normalization of transcripts was calculated after the geometric averaging of the reference genes. The relative transcripts of the target genes were determined with the 2^−ΔΔCT^ method [25]. A list of the primers of the genes that were evaluated, together with their Genbank accession numbers, is presented in Table 2.

### 2.5. Antibacterial Activity Experiments

Antibacterial activity experiments were performed as described in our recent publication [19]. Larval hemolymph was isolated 24 h after the RNAi treatment. The hemolymph was boiled at 100 °C for 5 min and centrifuged at 4 °C at 10,000 rpm for 10 min, and the supernatant was collected and stored at −20 °C [26].

For the bacterial growth curve experiment, *E. coli* and *S. aureus* were cultured in LB medium at 37 °C with shaking at 200 rpm until an OD_600_ = 0.3 was achieved. A total of 80 µL of bacterial culture was mixed with 20 µL of heat-treated hemolymph to a final volume of 100 µL in a 96-well plate. The plate was incubated at 37 °C and the OD_600_ was measured every hour with an Infinite M Plex microplate reader (Tecan, Männedorf, Switzerland).

For the inhibition zone experiment, LB agar medium was melted by heating and then cooled to approximately 50 °C. *E. coli* and *S. aureus* (OD_600_ = 0.3) were added to the medium at ratios of 1:100 and 1.5:100, respectively, mixed well, and poured into Petri dishes. Filter paper disks of ∼6 mm in diameter were placed on the agar medium, and 20 μL of heat-treated hemolymph was spotted on the filter paper disk. A total of 20 μL of sterile water or ampicillin (20 ng/μL) was added separately as a negative or positive control. The Petri dishes were incubated overnight in an incubator at 37 °C. The diameter of the inhibition zone of bacterial replication was observed the next day.

### 2.6. Data Analysis

Statistical analysis was performed using one-way ANOVA using SPSS version 26.0. Data were plotted in GraphPad Prism v10.0 and expressed as the mean ± standard deviation of replicates from three biological replications. The knockdown rate was compared to the control using the mean values and converted into percentages.

## 3. Results

### 3.1. The Silencing of BmToll9-2 Reduced the Expression of Signaling Genes in the Toll Pathway

**Figure 1:**
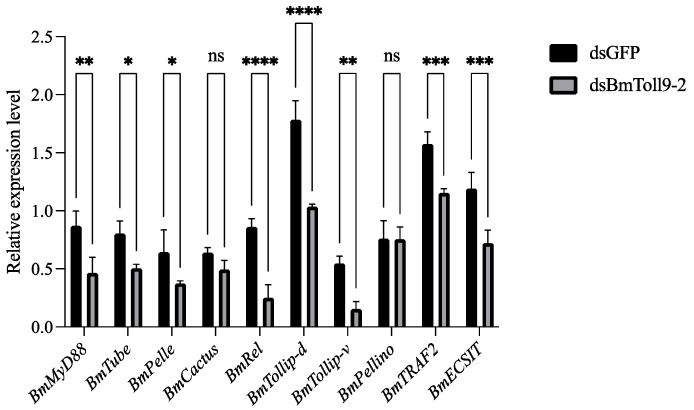
The relative expression of signaling genes in the Toll pathway after the RNAi of *BmToll9-2*. Larvae of the 5th instar were injected with dsBmToll9-2, and dsGFP served as a control. Data are represented as the means ± standard deviations of three biological replications. Asterisks indicate significant differences from dsGFP injection groups: * *p* < 0.05; ** *p* < 0.01; *** *p* < 0.001; and **** *p* < 0.0001. ns, not significant.

Our recent publication indicated that the RNAi of *BmToll9-2* was the most effective in the midgut, with a 69.73% knockdown after 24 h [20]. To evaluate whether the silencing of *BmToll9-2* affected components in the Toll pathway, downstream signaling genes in the midgut tissues were detected after the RNAi of *BmToll9-2* (Figure 1). Compared to the control group (injection with dsGFP), the signaling genes in the Toll pathway, except *BmCactus* and *BmPellino*, were significantly down-regulated in the RNAi group (injection with dsBmToll9-2). *BmMyD88*, *BmTube*, *BmPelle*, and *BmRel* were significantly inhibited by 46.55%, 37.27%, 41.86%, and 70.93%, respectively. *BmTollip-d*, *BmTollip-v*, *BmTRAF2*, and *BmECSIT* were significantly suppressed by 42.02%, 71.56%, 26.67%, and 39.50%, respectively.

### 3.2. Silencing of BmToll9-2 Reduced Expression of Downstream Effector Genes

**Figure 2:**
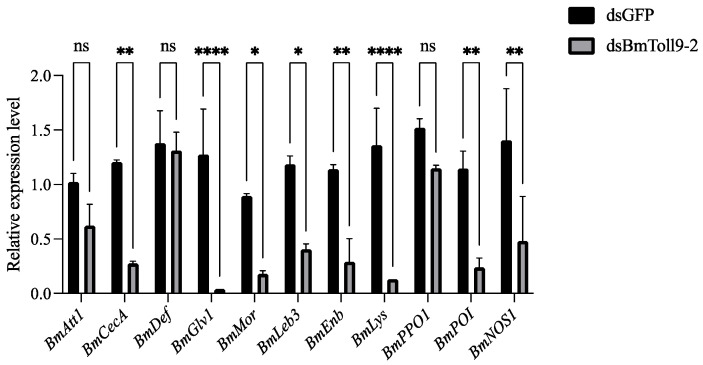
The relative expression of immune effector genes after the RNAi of *BmToll9-2*. Larvae of the 5th instar were injected with dsBmToll9-2, and dsGFP served as a control. Data are represented as the means ± standard deviations of three biological replications. Asterisks indicate significant differences from dsGFP injection groups: * *p* < 0.05; ** *p* < 0.01; and **** *p* < 0.0001. ns, not significant.

Similarly, midgut samples were used to detect the expression of 11 effector genes (Figure 2). Compared to the control group, most AMP genes were down-regulated in the RNAi group. The *Cecropin* gene, *BmCecA*, was significantly down-regulated by 77.18%. The *Gloverin* gene, *BmGlv1*, was significantly decreased by 96.86%. The *Moricin* gene, *BmMor*, showed a significant 79.89% reduction. The *Lebocin* and *Enbocin* genes, *BmLeb3* and *BmEnb*, were significantly reduced by 65.82% and 74.56%, respectively. The *Attacin* gene, *BmAtt1*, was down-regulated, although the differences were not statistically different. The *Defensin* gene, *BmDef*, showed no significant differences in both groups.

For the other effector genes, the *Lysozyme* gene, *BmLys*, was significantly reduced by 90.44%. The prophenoloxidase (*BmPPO1*) and phenoloxidase inhibitor (*BmPOI*) genes, involved in melanization, were down-regulated, but only *BmPOI* was significantly down-regulated by 90.44%. The gene encoding nitric oxide synthase, *BmNOS1*, was significantly reduced by 65.84%.

### 3.3. Silencing of BmToll9-2 Decreased Antibacterial Activity

To further investigate whether the silencing of *BmToll9-2* affected the humoral immune response in the silkworm larvae, hemolymph was collected for antibacterial activity assays against the Gram-negative bacterium *E. coli* and the Gram-positive bacterium *S. aureus*.

**Figure 3:**
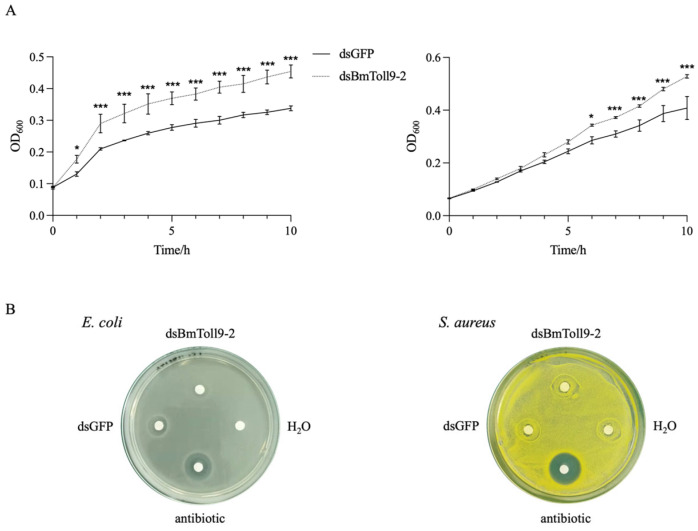
Antibacterial activity assays of *B. mori* hemolymph against *E. coli* and *S. aureus* after RNAi of *BmToll9-2.* Hemolymph was collected 24 h after dsRNA injection and tested for antibacterial activity. (**A**) Bacterial growth curve experiment. (**B**) Inhibition zone experiment. dsBmToll9-2: hemolymph from larvae injected with dsBmToll9-2; dsGFP: hemolymph from larvae injected with dsGFP; H_2_O: sterile water; antibiotic: ampicillin. Asterisks indicate significant differences from dsGFP injection groups: * *p* < 0.05 and *** *p* < 0.001.

The *E. coli* bacterial growth curve experiment showed that in the presence of hemolymph from the *BmToll9-2*-silenced larvae, the bacteria grew better than in the presence of hemolymph from the control group, with significant differences between 2 and 10 h (Figure 3A). In the inhibition zone experiment, both ampicillin and dsGFP-injected hemolymph showed antibacterial activity in the *E. coli* plate, while sterile water and dsBmToll9-2-injected hemolymph displayed no inhibitory effects (Figure 3B).

As for *S. aureus*, the dsBmToll9-2-injected hemolymph showed a significant difference in the bacterial growth curve after 6 h (Figure 3A). However, the dsBmToll9-2-injected hemolymph showed no significant differences in the inhibition zone experiment (Figure 3B). The above results indicated that the hemolymph of the silkworm larvae lost antibacterial activity when *BmToll9-2* was silenced, which might preferentially act against Gram-negative bacteria.

### 3.4. Bacterial Challenges Following the RNAi of BmToll9-2 Induced Signaling Genes in the Toll Pathway

**Figure 4:**
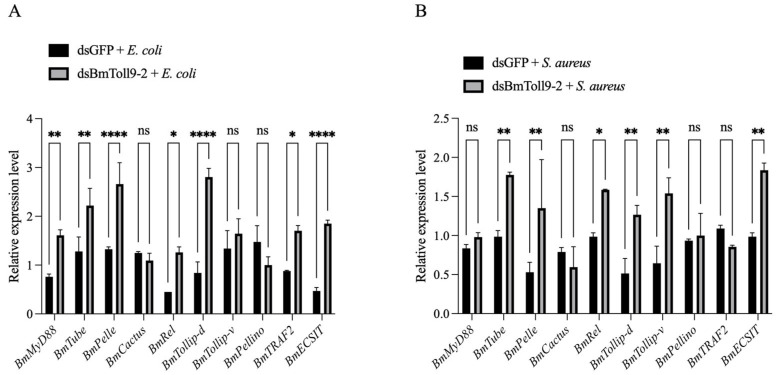
The relative expression of signaling genes in the Toll pathway following challenges with heat-inactivated bacteria after the RNAi of *BmToll9-2*. Larvae of 5th instar were injected with dsBmToll9-2 or dsGFP. Then, the larvae were fed with heat-killed (**A**) *E. coli* or (**B**) *S. aureus.* Data are represented as the means ± standard deviations of three biological replications. Asterisks indicate significant differences from dsGFP injection groups: * *p* < 0.05; ** *p* < 0.01; and **** *p* < 0.0001. ns, not significant.

In our recent report, bacterial challenges by the feeding of *E. coli* and *S. aureus* induced the expression of *BmToll9-2* in multiple tissues. Bacterial challenges following RNAi up-regulated the expression of *BmToll9-2* in the midgut [19]. This phenomenon was interpreted as a “hyper-induction” following a positive feedback mechanism that reacted to the depletion of *BmToll9-2* by the RNAi. In order to further confirm the role of bacterial infection as a trigger between *BmToll9-2* and downstream genes in the Toll pathway, signaling genes were detected after bacterial challenges following the RNAi of *BmToll9-2* (Figure 4).

Compared to the control group, the feeding of heat-inactivated *E. coli* to larvae after the RNAi of *BmToll9-2* increased the relative expression of most signaling genes (Figure 4A). *BmMyD88*, *BmTube*, and *BmPelle* were significantly induced by 2.12, 1.73, and 2.01 folds, respectively. *BmRel* and *BmTollip-d* were significantly up-regulated by 2.80 and 3.34 folds. *BmTRAF2* and *BmECSIT* showed a significant induction by 1.94 and 3.94 folds, respectively. *BmCactus*, *BmTollip-v*, and *BmPellino* showed no significant differences between the two treatments.

Similarly, the feeding of heat-inactivated *S. aureus* to larvae following the RNAi of *BmToll9-2* up-regulated the relative expression of most signaling genes, though with a lower intensity (Figure 4B). *BmTube* and *BmPelle* were significantly induced by 1.80 and 2.55 folds, respectively. *BmRel*, *BmTollip-d*, and *BmTollip-v* were significantly up-regulated by 1.61, 2.46, and 2.39 folds. *BmECSIT* showed a significant induction by 1.86 folds. *BmMyD88*, *BmCactus*, *BmPellino*, and *BmTRAF2* showed no significant differences between the two treatments.

### 3.5. Bacterial Challenges Following RNAi of BmToll9-2 Induced Downstream Effector Genes

**Figure 5:**
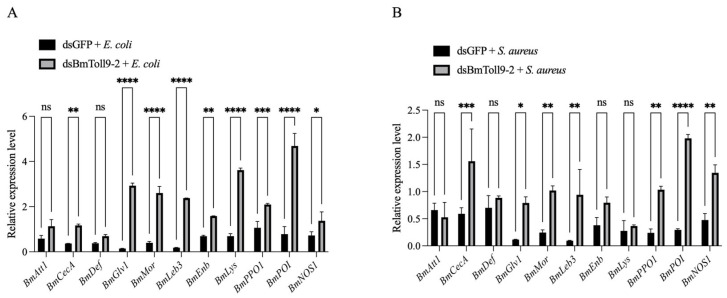
The relative expression of immune effector genes following challenges with heat-inactivated bacteria after the RNAi of *BmToll9-2*. Larvae of 5th instar were injected with dsBmToll9-2 or dsGFP. Then, the larvae were fed with heat-killed (**A**) *E. coli* or (**B**) *S. aureus*. Data are represented as the means ± standard deviations of three biological replications. Asterisks indicate significant differences from dsGFP injection groups: * *p* < 0.05; ** *p* < 0.01; *** *p* < 0.001; and **** *p* < 0.0001. ns, not significant.

In order to further verify the relationship between *BmToll9-2* and downstream effector genes, the same samples after bacterial challenges following the RNAi above were further investigated, which showed that the feeding of heat-inactivated bacteria induced most downstream effector genes (Figure 5).

In the treatment of heat-inactivated *E. coli* after the RNAi of *BmToll9-2*, most effector genes were induced (Figure 5A). The AMP genes *BmCecA*, *BmGlv1*, *BmMor*, *BmLeb3*, and *BmEnb* were significantly up-regulated in the RNAi group by 3.21, 20.93, 6.58, 13.19, and 2.29 folds, respectively. For the other effector genes, *BmLys*, *BmPPO1*, *BmPOI*, and *BmNOS1* were induced significantly by 5.24, 1.98, 6.01, and 1.90 folds, respectively. The relative expressions of *BmAtt1* and *BmDef* were not significantly different.

Similarly, feeding with heat-inactivated *S. aureus* following the RNAi of *BmToll9-2* increased the relative expression of most AMPs (Figure 5B). The relative expressions of *BmCecA*, *BmGlv1, BmMor*, and *BmLeb3* were significantly induced by 2.64, 6.87, 4.16, and 9.89 folds, respectively. The other effector genes, including *BmPPO1*, *BmPOI*, and *BmNOS1*, were up-regulated significantly by 4.31, 6.71, and 2.83 folds, respectively. Meanwhile, the relative expressions of *BmAtt1*, *BmDef*, *BmEnb*, and *BmLys* were not significantly different. It was noticed that *BmPPO1* and *BmNOS1* showed a higher induction fold after *S. aureus* than *E. coli*. This might have been due to the fact that the activation of PPO1 is important in survival during infection with Gram-positive bacteria [27]. The production of nitric oxide (NO) by NOS is effective in eradicating *Staphylococcus* bacteria [28].

In general, after treatment with heat-inactivated bacteria, the expression pattern of most AMPs and other effector genes was positively correlated with *BmToll9-2*, while the induction of *E. coli* was stronger than that of *S. aureus*.

## 4. Discussion

Toll9 is a conserved membrane receptor in insects. Previous reports have shown that Toll9 mainly functions in immunity [19]. In our recent report, *BmToll9-2* was characterized for the first time through a reverse functional study, which showed that *BmToll9-2* was activated by the Gram-negative bacterium *E. coli* and its cell wall component, LPS. The loss of *BmToll9-2* may imbalance gut homeostasis, resulting in a defect in the growth of larvae [20]. However, whether *BmToll9-2* is involved in regulating the Toll signaling pathway remains to be answered. In this study, we investigated the role of *BmToll9-2* as a regulator in the Toll signaling pathway and downstream effector genes and as a modulator in the humoral immune response in antibacterial activity in hemolymph.

### 4.1. BmToll9-2 Is Involved Positively in the Toll Pathway

MyD88, Tube, Pelle, Cactus, and Rel are the intermediary factors of the Toll pathway in the cytoplasm. Intracellular components such as Tollip, Pellino, TRAF2, and ECSIT are also factors involved in this process [8,29]. In this study, the silencing of *BmToll9-2* through RNAi resulted in the significant knockdown of the above signaling genes except *Cactus* and *Tollip-v* (Figure 1). *BmToll9-2* was found to be induced by bacterial challenges, especially with the Gram-negative bacterium *E. coli*, either before or after the RNAi of *BmToll9-2* [20]. In the latter case, the silencing of *BmToll9-2* may have triggered a feedback mechanism that restored its expression levels to high levels following bacterial challenges [20]. Therefore, the same samples treated with bacterial challenges following RNAi were tested, which showed a significant up-regulation of most signaling genes in either the *E. coli* or *S. aureus* treatment (Figure 4). This indicates that when *BmToll9-2* was induced, most signaling genes were also activated.

The relation of *Toll* genes to factors in the immune signaling pathway is extensively studied. In our previous study, the over-expression of *BmToll9-1* suppressed signaling genes in the IMD and Jak/Stat pathways after treatment with LPS [14]. On the other hand, *BmToll9-1* regulated the expression of signaling genes in the Toll pathway positively [19], which was consistent with observations of other species. In the Chinese oak silkworm, *Antheraea pernyi*, the up-regulation of the Toll gene by a fungus resulted in the induction of the *MyD88*, *Cactus*, and *Rel* genes in the Toll pathway [30]. In the honey bee, the up-regulation of the MyD88 gene was associated with the induction of the Toll gene [31]. In the small brown planthopper, *Laodelphax striatellus*, the rice stripe virus activated the Toll pathway by inducing the expression of the *Tube, MyD88*, and *Dorsal* genes [32].

### 4.2. BmToll9-2 Positively Regulates Immune Effectors

Immune effectors, including AMPs, directly interact with infectious pathogens to cause their neutralization [8]. When *BmToll9-2* was silenced, most AMPs and other immune effectors were suppressed (Figure 2). Meanwhile, hemolymph from *BmToll9-2*-silenced larvae showed decreased antibacterial activity against *E. coli* and *S. aureus*, either in the growth curve or inhibition zone experiments (Figure 3). The up-regulation of *BmToll9-2* after bacterial challenges following RNAi also induced most AMPs and other immune effectors (Figure 5). These results suggest that *BmToll9-2* expression is positively correlated to the production of immune effectors.

Studies on Toll receptors to regulate AMP effectors have been reported before. We previously found that in silkworm-derived Bm5 cells over-expressing *BmToll9-1*, the soaking of LPS suppressed the transcription of *BmToll9-1* and AMPs [14]. We recently reported that the silencing of *BmToll9-1* resulted in the down-regulation of most immune effectors in silkworm larvae [19]. The above results suggest that *BmToll9-1* acts as a positive regulator of AMPs and other immune effectors. Since *BmToll9-1* and *BmToll9-2* are phylogenetically close to each other, it is reasonable to see their similar role in positively regulating immune effectors. In addition, the silencing of *BmToll9-1* decreased antibacterial activity [19], which also occurred after the silencing of *BmToll9-2*.

The regulation of AMPs by other receptors has also been reported in the silkworm extensively. For example, we reported that BmPGRP-L4 negatively regulated the production of AMPs [26]. BmPGRP-S5 was also reported as a negative regulator in AMP production [33]. As part of the insulin-like signaling pathway, AMPs were induced after starvation [34]. The regulation of AMPs by scavenger receptor C was achieved by activating the Toll signaling pathway [35].

### 4.3. BmToll9-2 Is Preferentially Activated by Gram-Negative Bacteria

Previously, we reported that the Gram-negative bacterium *E. coli* and its main cell wall component, LPS, remarkably induced the expression of *BmToll9-2* in silkworm larvae and in silkworm-derived BmN4 cells [20]. In this study, the induction fold of *E. coli* in the expression of AMPs was higher than that of *S. aureus* (Figure 5). Meanwhile, the decrease in antibacterial activity against *E. coli* compared to *S. aureus* after RNAi was even more obvious (Figure 3).

A similar response to *E. coli* was also described in our recent publication investigating the role of *BmToll9-1*. When *BmToll9-1* was silenced, the hemolymph of silkworm larvae lost antibacterial activity against the Gram-negative bacterium *E. coli* but not against the Gram-positive bacterium *S. aureus* [19]. Interestingly, *BmToll9-2* is phylogenetically related to TLR4 [20], the PRR for LPS recognition in mammals [36]. A recent report also proved that *BmToll9-1* functions as a PRR that recognizes LPS, similarly to TLR4 [18]. Considering its level of similarity and the phylogenetic analysis, *BmToll9-2* might also be a candidate PRR to detect LPS. Therefore, it is reasonable that *BmToll9-2* is prone to being triggered by *E. coli*.

## 5. Conclusions

The role of *BmToll9-2* in regulating the expression of downstream elements in the Toll pathway was investigated in this study. The silencing of *BmToll9-2* reduced the expression of signaling and downstream effector genes in the Toll signaling pathway. Antibacterial activity in the hemolymph, especially against *E. coli*, was also decreased. The induction of *BmToll9-2* after bacterial treatment following RNAi also up-regulated both the signaling genes in the Toll pathway and its downstream effector genes. The above results suggest that, similarly to *BmToll9-1*, *BmToll9-2* might positively regulate the Toll signaling pathway, which in turn stimulates the humoral immune response and antibacterial activity.

## Figures and Tables

**Table 1:** Current studies on Toll receptor genes in *Bombyx mori*.

Year	Genes	Summary of Paper and Reference
2002	BmToll7	The molecular cloning and expression of BmToll7, which was suppressed by LPS [11].
2008	13 *Bombyx mori* Tolls	The identification and analysis of Toll-related genes in the domesticated silkworm was performed for the first time [10].
2008	14 *Bombyx mori* Tolls	A genome-wide analysis of genes and gene families involved in the innate immunity of *Bombyx mori* was conducted, including 14 Toll-related genes [8].
2010	*BmToll9-1*	BmToll9 is probably involved in the local gut immune response in the silkworm [12].
2013	*BmToll9-1*	Both LPS and dsGFP, by injection, significantly inhibited the transcription of *BmToll9-1* in silkworm larvae [13].
2014	*BmToll9-1*	*BmToll9-1* repressed the IMD and Jak–STAT pathway genes and AMP effector genes by LPS [14].
2016	8 *Bombyx mori* Tolls	The expression levels of eight Toll genes were altered by challenging them with *Beauveria bassiana*. Toll signaling pathway inhibitors significantly inhibited the anti-fungal activity in hemolymph and resulted in the increased sensitivity of silkworm larvae to *Beauveria bassiana* infection [15].
2019	8 *Bombyx mori* Tolls	The role of Toll receptors in the inhibition of BmNPV proliferation was confirmed [16].
2020	14 *Bombyx mori* Tolls	Spätzle2 (BmSpz2) could be activated by pathogens. Activated BmSpz2 could bind with BmToll11 or *BmToll9-1* [17].
2021	*BmToll9-1*	*BmToll9-1* is a pattern-recognition receptor for LPS that shares conserved features with the mammalian TLR4–MD-2–LPS pathway [18].
2024	*BmToll9-1*	*BmToll9-1* is a positive regulator of the immune response in the silkworm, *Bombyx mori* [19].
2024	*BmToll9-2*	*BmToll9-2* was preferentially triggered by *Escherichia coli* and LPS. The RNAi of *BmToll9-2* reduced the weight and growth of silkworm larvae [20].

**Table 2:** A list of primers used in this study.

Gene	Accession Number	Primer Sequence (5′–3′)
Primers for qPCR		
BmTIF4A	DQ443290	F: TTCGTACTGGCTCTTCTCGT
		R: CAAAGTTGATAGCAATTCCCT
BmTIF3s4	DQ443238	F: ACTTCAAGTTCAGGGCAGAT
		R: TTAATTGTTTTGTGGAGGCT
Signaling		
BmMyD88	XM_028186400	F: AACGGTCACGACTCGAACTC
		R: TCTGCCCAGATTCTTCATCC
BmTube	XM_028173146	F: GGCAGAAAGTTATGGCTTGG
		R: ATCCTCAAATGCTCGCTGTT
BmPelle	XM_028182154	F: ACATCAAGCCGGCTAACATC
		R: ACCGTGAGACCTTCAGATGC
BmCactus	XM_028180230	F: ACAGTCGTGCGTACATTTGG
		R: CAGCCTCTCCCTATCGTCAA
BmRel	XM_028175224	F: TCGAATACATCCCGGACTTC
		R: TGGAAGGTCCTTTCTTGCTC
BmTollip-d	XM_021351983	F: GACGAGTCAGTCCCTCTTGC
		R: GTGGCTGGTGGAATTCGTAG
BmTollip-v	XM_028186930	F: TGCTACTTCTGACGGTGTGG
		R: AGGGCCACTTTGTGGTACTG
BmPellino	XM_028184930	F: AGAGTCGCTCAGCACAACAA
		R: CAATGTGGCTCCACACAGAT
BmTRAF2	XM_028172769	F: TCGCTCCTATGGGCATAACT
		R: CCGCATGTTGTGATTACTGG
BmECSIT	XM_028171307	F: ATGCCGCCTTAGCTAGAATG
		R: GCCTTTGGGCAGTACGTCTA
Effectors		
BmAttacin1 (BmAtt1)	NM_001043541	F: CAGTGAACTCGGATGGAACC R: GGCGCTGAGTACGTTCTTGT
BmCecropinA (BmCecA)	NM_001043997	F: CCGTCATAGGGCAAGCGAAA R: AGCAATGACTGTGGTATGTCAA
BmDefensin (BmDef)	AB_367525	F: GTTAAGTGCGGCGTTGACTG R: TGACAGGGAAAGTGGAAGGG
BmGloverin1 (BmGlv1)	AB_289654	F: GCTGGGATAGAAGCATCAGC R: ACATCAGGCCTTCTGTGACC
BmMoricin (BmMor)	AB_006915	F: TGTGGCAATGTCTCTGGTGT R: CTGGCGATATTGATGGCTCT
BmLebocin3 (BmLeb3)	NM_001126260	F: CTCGATCCAAACCGAAGGTA R: CGGCTGGTCAAGTCCAGTAT
BmEnbocin (BmEnb)	FJ373019	F: ACCTCGCACAACTAGTTCGG R: CCAACAGAACAAACCCACTCG
BmLysozyme (BmLys)	NM_001043983	F: TAACGGCTCGAAGGACTACG R: GAGGTCGGAGCACTTAACGT
Phenoloxidase inhibitor (BmPOI)	XR_001139981	F: GGATACGTGACTGGAAATGCA R: GTCATAATCCACGGGTTTGTCC
Prophenoloxidase 1 (BmPPO1)	AF_178462	F: AGTGGGAAGCCATTCTCCTT R: GCCAGGTTTCACTCCTTGAG
Nitric oxide synthase 1 (BmNOS1)	XM_012689821	F: TCATCACCACTAGCGCATCC R: CCTTGTCCGTTCTGTGTCCT
Primers for dsRNA synthesis		
T7-BmToll9-2	PP716770	F: TAATACGACTCACTATAGG TAGTATTCTCCCGGCTCTC
		R: TAATACGACTCACTATAGG GAAGGGTGCCTTGTGTAATC
T7-GFP	GQ357182	F: TAATACGACTCACTATAGG TACGGCGTGCAGTGCT
		R: TAATACGACTCACTATAGG TGATCGCGCTTCTCG

## Data Availability

The raw data supporting the conclusions of this article will be made available from the corresponding author upon reasonable request.
